# Trends in glyphosate herbicide use in the United States and globally

**DOI:** 10.1186/s12302-016-0070-0

**Published:** 2016-02-02

**Authors:** Charles M. Benbrook

**Affiliations:** Benbrook Consulting Services, 90063 Troy Road, Enterprise, OR 97828 USA

**Keywords:** Glyphosate, Herbicide use, Genetic engineering, Herbicide-tolerant crops, Roundup, Pesticide use

## Abstract

**Background:**

Accurate pesticide use data are essential when studying the environmental and public health impacts of pesticide use. Since the mid-1990s, significant changes have occurred in when and how glyphosate herbicides are applied, and there has been a dramatic increase in the total volume applied.

**Methods:**

Data on glyphosate applications were collected from multiple sources and integrated into a dataset spanning agricultural, non-agricultural, and total glyphosate use from 1974–2014 in the United States, and from 1994–2014 globally.

**Results:**

Since 1974 in the U.S., over 1.6 billion kilograms of glyphosate active ingredient have been applied, or 19 % of estimated global use of glyphosate (8.6 billion kilograms). Globally, glyphosate use has risen almost 15-fold since so-called “Roundup Ready,” genetically engineered glyphosate-tolerant crops were introduced in 1996. Two-thirds of the total volume of glyphosate applied in the U.S. from 1974 to 2014 has been sprayed in just the last 10 years. The corresponding share globally is 72 %. In 2014, farmers sprayed enough glyphosate to apply ~1.0 kg/ha (0.8 pound/acre) on every hectare of U.S.-cultivated cropland and nearly 0.53 kg/ha (0.47 pounds/acre) on all cropland worldwide.

**Conclusions:**

Genetically engineered herbicide-tolerant crops now account for about 56 % of global glyphosate use. In the U.S., no pesticide has come remotely close to such intensive and widespread use. This is likely the case globally, but published global pesticide use data are sparse. Glyphosate will likely remain the most widely applied pesticide worldwide for years to come, and interest will grow in quantifying ecological and human health impacts. Accurate, accessible time-series data on glyphosate use will accelerate research progress.

**Electronic supplementary material:**

The online version of this article (doi:10.1186/s12302-016-0070-0) contains supplementary material, which is available to authorized users.

## Background

A Swiss chemist working for a pharmaceutical company, Dr. Henri Martin, discovered glyphosate [N-(phosphonomethyl) glycine] in 1950 [1]. Because no pharmaceutical applications were identified, the molecule was sold to a series of other companies and samples were tested for a number of possible end uses. A Monsanto chemist, Dr. John Franz, identified the herbicidal activity of glyphosate in 1970, and a formulated end-use product called Roundup was first sold commercially by Monsanto in 1974 [2].

For two decades, the number and diversity of agricultural and non-farm uses grew steadily, but the volume sold was limited because glyphosate could only be sprayed where land managers wanted to kill all vegetation (e.g., between the rows in orchards and viticulture; industrial yards; and, train, pipeline, and powerline rights of way). Some applications were, and still are made after a crop is harvested, to control late-season weeds that escaped other control measures. In some regions, desiccant applications are made late in the growing season to speed up harvest operations, especially in small grain crops.

In 1996, so-called “Roundup Ready” (RR), genetically engineered (GE) herbicide-tolerant (HT) soybean, maize, and cotton varieties were approved for planting in the U.S. This technological breakthrough made it possible to utilize glyphosate as a broadcast, post-emergence herbicide, thereby dramatically extending the time period during which glyphosate-based herbicides could be applied. Alfalfa and sugar beets engineered to tolerate glyphosate were first approved and commercially marketed in 2005 and 2008, respectively, but federal lawsuits citing procedural violations of the National Environmental Policy Act delayed full commercial sales until 2011 for RR alfalfa and 2012 for RR sugar beets [3, 4].

To quantify the environmental and human health impacts stemming from pesticide use, it is essential to know how much pesticide is being applied in a region on a given crop, collectively across all crops, and in other places (e.g., forests, rangeland, along rights-of-way, industrial yards). Ideally, data are available on the land area and crops treated; the timing and method of applications; rates and number of application; the formulation applied and the total volume applied per hectare. Unfortunately, all these data are rarely available.

*Rising use heightens risk concerns*. Growing reliance on the broad-spectrum herbicide glyphosate has triggered the spread of tolerant and resistant weeds in the U.S. and globally [5–10]. To combat weeds less sensitive to glyphosate, farmers typically increase glyphosate application rates and spray more often [11–13]. In addition, next-generation herbicide-tolerant crops are, or will soon be on the market genetically engineered to withstand the application of additional herbicides (up to over a dozen), including herbicides posing greater ecological, crop damage, and human health risks (e.g., 2,4-D and dicamba) [6].

This paper presents trends in glyphosate use in order to help researchers better understand and quantify the risks and benefits stemming from uses of glyphosate-based herbicides. Detailed data on trends in glyphosate use in the U.S., both in and outside the agricultural sector, are presented, while the data on global glyphosate use are less complete and more uncertain. Fortunately, sufficient data are available to track the impact of GE herbicide-tolerant (HT) crops on global glyphosate-based herbicide (GBH) use since 2010 [14–17].

**Fig. 1:**
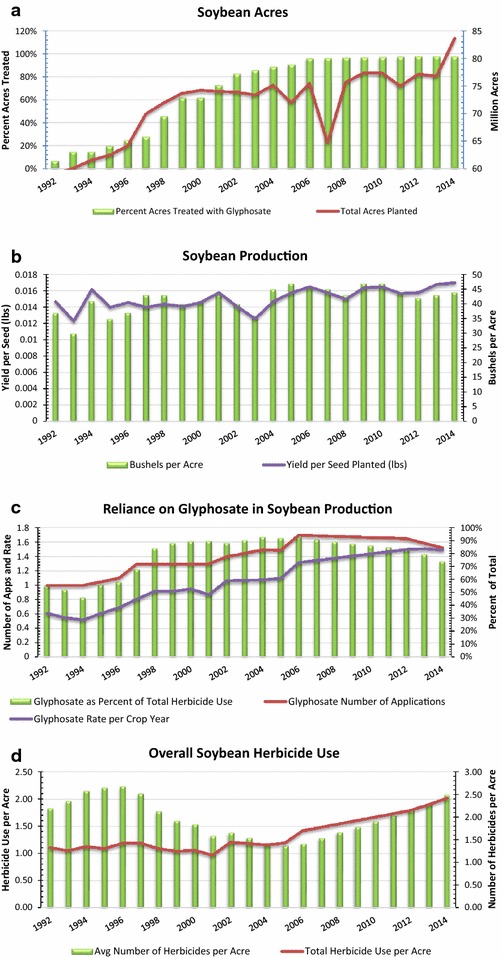
Trends in U.S. soybean production and glyphosate use

In order to better understand the many factors driving the trajectory of glyphosate’s use and impacts, two timeline graphics are presented in the “Discussion” section, Fig. 1.

## Methods

### Use data

Throughout this paper, all references to glyphosate or glyphosate-based herbicides encompass all commercial end-use formulations. All data on volumes of glyphosate applied refer to kilograms or pounds of the active ingredient glyphosate, rather than glyphosate plus the adjuvants and surfactants included in an end-use formulation to enhance uptake by weeds and facilitate mixing and spray applications.

Glyphosate is applied in a variety of forms including isopropylamine salt, ammonium salt, diammonium salt, dimethylammonium salt, and potassium salt [1]. E.g., in its corn pesticide use survey in 2014, the National Agricultural Statistics Service (NASS) collected data on four different forms of glyphosate applied at different rates: isopropylamine salt, glyphosate, glyphosate ammonium salt, and glyphosate potassium salt [11]. Total corn hectares treated with glyphosate in the U.S. and kilograms of active ingredient applied are the sum across the four forms of glyphosate. “Total glyphosate” rate of application is calculated as an average of the four application rates reported for the different forms of glyphosate, weighed by the area treated with each form of glyphosate. The same process can be used to calculate “total glyphosate” average number of applications per hectare.

Four data points are generally collected and/or calculated when government agencies or private survey companies report pesticide use data on a given crop in a defined area and time period: (1) the percent of crop hectares treated with a given pesticide; (2) the average rate of application; (3) the average number of applications per crop year; and (4) total kilograms of pesticide applied to the crop. When a data source does not report total kilograms or pounds applied, or one other of the above key parameters, the missing variable can be calculated based on:

1 $$Weight_{p,c} = \frac{{Area Treated_{p,c} }}{{Total Area_{c} }} \times Total Area_{c} \times Rate_{p,c}$$

where *Weight*_*p*,*c*_ is the amount of pesticide *p* applied to crop *c* (kg active ingredient [a.i.]), *AreaTreated*_*p,c*_ is the area of crop *c* to which the pesticide *p* is applied (ha), *TotalArea*_*c*_ is the total area planted with crop *c* (ha), and *Rate*_*p*,*c*_ is the “Rate per Crop Year” for pesticide *p* on crop *c*. “Rate per Crop Year” is the product of the average rate of application multiplied by the number of applications per crop year, and is a useful metric because certain crops may be planted in the fall and harvested the next spring or summer of the following year.

### U.S. data sources

The U.S. Department of Agriculture (USDA), through the National Agricultural Statistics Service (NASS), has collected reasonably comprehensive pesticide use data for major grain, row crop, fruit, and vegetable crops since 1990 [18]. Periodic USDA surveys are also available to track pesticide use on major crops back into the 1970s.

Between 1997 and 2007, the U.S. Environmental Protection Agency (EPA) issued several reports on the volumes of pesticides applied in the agricultural, industrial/government, and urban/suburban sectors [19–23]. EPA use reports capture a number of lower-volume pesticide uses not included in USDA surveys and are the only public source of data on industrial/government and suburban/urban pesticide use.

Data on glyphosate use on specific crops in the U.S. are primarily drawn from pesticide use surveys carried out by the USDA’s NASS. Pesticide applications at the national and state level have been reported since 1990 by NASS for most major field crops; fruit crops have been surveyed in odd years; and vegetables have been covered in even years [18].

Estimates of overall GBH use by U.S. farmers and ranchers are available from three sources: the sum of crop-specific NASS data in any given year; the EPA periodic reports noted above; and, the pesticide use data set compiled by the U.S. Geological Survey (USGS), which in turn draws heavily on private survey data [24–26]. Both the EPA and USGS use data compilations augment NASS data with a variety of other information sources that cover uses not included in NASS surveys. In addition, a number of private companies conduct surveys of pesticide use in the U.S. and around the world, although detailed results are not publicly accessible.

NASS surveys a limited number of crops in any given year. In the tables that follow, pesticide use is linearly interpolated in years lacking survey data but bounded by reported values. In years before the first, or after the last NASS survey, annual values are extrapolated (see [27], Additional file 1: Tables for details).

In each year, NASS strives to collect data on states that collectively account for at least 85 % of the area planted nationally to a given crop. For some crops, 15 or more states are surveyed to reach this threshold, while in other crops only two states are required (e.g., lemons in 2011, two states; corn in 2010, 21 states). Accordingly, when NASS reports national estimates of total pesticide use on *surveyed* acres of a given crop, the data typically underestimate total national crop use by ~15 %, since national acres planted always exceeds NASS acres surveyed. This is why in several Additional file 1: Tables [27] the pounds of herbicides applied are reported on both NASS-surveyed acres and total national acre. To estimate use on all planted hectares/acres, the average rate of application per crop year on NASS-surveyed acres is applied to the total planted area [28].

### Total volume of glyphosate applied

NASS use data were downloaded and integrated into the “Pesticide Use Data System” (PUDS). Additional file 1: Tables S6–S15 [27] report glyphosate use in the U.S. on grain crops, fruits, vegetables, nuts, and other crops for 1982, 1992, 1995, 1998, 2001, 2004, and every 2 years thereafter through 2014. These tables report average rates of glyphosate application and rate per crop year weighted by the acres treated with each of the multiple forms of glyphosate included in NASS surveys. Total pounds of all forms of glyphosate applied to all crops surveyed by NASS are shown in Additional file 1: Table S17 [27]. Values in years when NASS did not survey a given crop are interpolated or extrapolated (see Additional file 1: Table S17 for details).

Little or no government or published survey data are accessible on the volume of glyphosate applied on canola and pima cotton, as well as two more recently approved and planted GE-HT crops (alfalfa, sugar beets). Estimates of GBH use on these crops were made for 2012–2014 in Additional file 1: Table S16, based on NASS data on acres planted, estimates of adoption of glyphosate-tolerant varieties issued by commodity groups, academic weed management specialists, or in trade press articles.

### EPA pesticide use summary reports

Pesticide use reports have been released by the EPA for 1987, 1993, and every two years thereafter through 2007 [19–23].

These reports encompass more crops and agricultural uses than the NASS reports, and also quantify use in three sectors: “U.S. Agriculture,” “Industrial/Commercial/Government,” and “Home and Garden.” EPA pesticide use reports draw on NASS survey results, a number of proprietary pesticide use datasets, and pesticide production and use data submitted by registrants, or collected during the course of a regulatory review of a given pesticide.

The EPA has not reported pesticide use data since 2007. However, NASS coverage of the major uses of glyphosate is somewhat consistent since 2007, and the U.S. Geological Survey (USGS) has also issued detailed reports and a dataset of pesticide use covering 1992-2011 [25, 29]. Results from NASS, EPA, and USGS are integrated in Additional file 1: Table S18 [27] to produce annual data from 1974 through 2014 in glyphosate use in agriculture, non-agricultural applications, and total glyphosate use.

*Global glyphosate use data sources and estimates.* A special issue of the journal *Pest Management Science* in 2000 focused on glyphosate uses, issues, and challenges. Woodburn [29] summarized global glyphosate use from 1994–1997, and provided valuable information on agricultural and non-agricultural uses. Woodburn’s analysis drew upon proprietary data sources and surveys.

Several sources of industry data on global glyphosate production are available for 2011–2014 [30–33]. Global use data in Additional file 1: Table S23 between 1997 and 2011 are interpolated and track the annual rates of growth in the U.S.

### Glyphosate use on herbicide-tolerant hectares

Global soybean production in 2014 was 315.4 million metric tons (11.6 million bushels), with the U.S. (108 million metric tons), Brazil (94.5 mill. metric tons), and Argentina (56 mill. metric tons) accounting for 82 % of the global harvest [34]. The International Service for the Acquisition of Agri-Biotech Applications (ISAAA) compiles annual, global data by country, continent, and worldwide on hectares planted to various GE crop varieties ([14–17], [27], Additional file 1: Table S20). Data from these briefs were combined with estimates of average glyphosate rates of application ([27], Additional file 1: Table S21), yielding estimates of total glyphosate use from 1996 to 2014 on GE, herbicide-tolerant cotton, maize, soybeans, and canola, and globally for all crops ([27], Additional file 1: Table S23).

*Use in Argentina and Brazil.* GE-HT soybeans accounted for 100 and 93 % of the soybean hectares planted in Argentina and Brazil in 2014 [34]. Sistema Integrado de Información Agropecuaria (Ministerio de Agricultura Ganadaria y Pesca) reports data on hectares planted to soybeans in Argentina [35], and the Instituto Brasileiro de Geografia e Estatística (IBGE) provides the same data for Brazil [36]. For Argentina and Brazil, Soystats [34] provides percent of area planted to GE-HT soybeans for 2000–2014. Benbrook [37] and Meyer and Cederberg [38] provide information on glyphosate use rates per crop year, which are substantially higher than those in the U.S. Ferraro and Ghersa [39] also document applications to soybeans in Argentina that can range up to seven per year, substantially more than in the U.S.

## Results

### Glyphosate use in the U.S

Farmers and ranchers in the U.S. applied an estimated 0.36 million kg of active ingredient (0.8 million pounds) in 1974 (Table 1). The volume applied increased steadily, but not dramatically, through 1995, to 12.5 million kg (28 million pounds).

**Table 1:** Glyphosate active ingredient use in the United States: 1974–2014

	1974	1982	1990	1995	2000	2005	2010	2012	2014
Glyphosate Use (1000 kg)	635	3538	5761	18,144	44,679	81,506	118,298	118,753	125,384
Agricultural	363	2268	3357	12,474	35,720	71,441	106,963	107,192	113,356
Non-agricultural	272	1270	2404	5670	8958	10,065	11,335	11,562	12,029
Glyphosate use (1000 lb)	1400	7800	12,700	40,000	98,500	179,690	260,804	261,807	276,425
Agricultural	800	5000	7400	27,500	78,750	157,500	235,814	236,318	249,906
Non-agricultural	600	2800	5300	12,500	19,750	22,190	24,989	25,489	26,519
Share agricultural (%)	57.1	64.1	58.3	68.8	79.9	87.7	90.4	90.3	90.4
Share non-agricultural (%)	42.9	35.9	41.7	31.3	20.1	12.3	9.6	9.7	9.6

Data in thousands of kilograms or pounds of glyphosate active ingredient. From the National Agriculture Statistical Service pesticide use data and the Environmental Protection Agency pesticide industry and use reports (1995, 1997, 1999, 2001, 2007). See Additional file 1: Table S18 for details

The 12.5 million kg applied in 1995, prior to the GE era, made glyphosate the seventh most heavily applied pesticide in U.S. agriculture that year, according to the EPA ([27], Additional file 1: Table S19). The top-six pesticides applied by U.S. farmers and ranchers in 1995 included two herbicides mostly used on corn (#1 atrazine, and #2 metolachlor), three soil fumigants primarily applied on fruit and vegetable crops (#3-5, metam-sodium, methyl-bromide, dichloropropene), and one broad-leaf herbicide relied on in multiple cropping systems (#6, 2,4-D).

As GE-HT crops gained market share in 1996–2000, agricultural applications of glyphosate in the U.S. rose rapidly, reaching 36 million kg (79 million pounds) by 2000 (Table 1). That year, agricultural uses of glyphosate accounted for 80 % of total national use (in 1974, the agricultural share of total glyphosate use was about 60 %). A decade later in 2010, agriculture’s share had risen to 90 %. From 1974–2014, a total of 1.37 billion kg of glyphosate (3.0 billion pounds) was applied in the U.S. agricultural sector (Table 1).

Glyphosate use in the agricultural sector rose 300-fold from 1974 to 2014 (0.36–113.4 million kg; 0.8–250 million pounds). Non-agricultural uses rose less dramatically, by 43-fold in the same time period, because there were far fewer new, non-agricultural uses registered.

Glyphosate has been on the market as a herbicide for 42 years. In the first 31 of these years (1974–2004), U.S., farmers applied only ~27 % of the total volume (weight) of glyphosate applied since 1974. Nearly 67 % of total agricultural glyphosate use in the U.S. since 1974 has occurred in just the last 10 years (Table 2).

**Table 2:** Share of total glyphosate active ingredient use by decade in the U.S

	Total use (million kg)	Increase from previous period	Share of total use 1974–2014 (%)
1974	0.6	NA	0.0
1975–1984	26	25	1.6
1985–1994	77.1	51	4.8
1995–2004	433	356	26.9
2005–2014	1070	637	66.6
Total	1607		

Estimated from National Agriculture Statistical Service (NASS), USGS, and EPA data. See Additional file 1: Table S18 for details

### Use by crop in the U.S

Table 3 provides an overview of trends since 1990 in glyphosate applications on 12 major crops in the U.S. surveyed by NASS, as well as an estimate of use on all other crops. Soybeans accounted for about one-third of total agricultural glyphosate use in 1990, a share that rises to almost one-half by 2014 (Table 3). The three major GE-HT crops (soybeans, maize, cotton) accounted for ~200 million pounds of glyphosate use based on NASS data, or 80 % of total farm and ranch use in 2014 (249.9 million pounds; Table 3). USGS data for 2012 place total GBH use on the three GE-HT crops at 235 million pounds; the difference between NASS and USGS data arises from higher USGS estimates of use on corn and cotton.

**Table 3:** Glyphosate active ingredient applied to major crop in the U.S., 1990–2014

	1990	1995	2000	2005	2010	2014
Soybeans	2,663,000	7,628,350	43,870,826	72,043,130	107,697,606	122,473,987
Corn	880,066	2,620,860	4,779,306	25,587,085	69,494,324	68,949,452
Cotton, upland	192,429	1,013,052	10,145,096	16,308,461	17,815,794	17,421,787
Wheat, winter	331,758	239,051	1,702,193	5,045,592	13,922,880	12,353,488
Alfalfa	381,525	402,666	422,334	469,539	479,184	8,853,600
Wheat, spring (excl. durum)	90,659	416,744	1,892,420	2,203,603	4,128,957	4,217,788
Sorghum	236,305	751,913	1,540,931	2,652,943	3,924,301	4,178,573
Sugar beets	36,130	59,012	87,439	118,139	2,226,610	2,763,075
Canola	0	0	552,632	647,368	1,284,317	219,392
Oranges	885,201	1,149,594	1,487,882	1,898,798	1,631,050	1,683,156
Wheat, spring durum	75,308	199,483	450,635	444,785	1,190,234	1,201,807
Barley	13,168	45,563	248,554	658,954	996,626	1,064,160
Other crops	1,897,522	2,733,922	3,736,751	4,249,288	4,648,224	4,526,043
Total crops	7,683,070	17,260,209	70,916,999	132,327,684	229,440,109	249,906,307

Data are pounds of active ingredient applied

National Agriculture Statistical Service. See Additional file 1: Table S17 for details

Detailed glyphosate use data for NASS-surveyed crops are provided in Additional file 1: Tables S11–21 [27] for 1982, 1992, 1995, 1998, 2001, 2004, and every even year thereafter through 2014. In each table, the following crop groups are used: grains, fruits, vegetables, nuts, and other crops. For each crop and year, the data points include percent of acres treated, rate of application, number of applications, rate per crop year, pounds applied to surveyed acres and to total national crop acres. Additional file 1: Table S5 provides glyphosate herbicide data at the state level for winter wheat in Kansas from 1993–2012.

### Global glyphosate use

Worldwide glyphosate use was modest in the 1970s compared to the most heavily applied herbicides then on the market (e.g. atrazine, metolachlor). The volume applied grew relatively slowly until the GE era ([27], Additional file 1: Table S24). By 1994, global agricultural use had reached 43 million kg of active ingredient (95 million pounds). Another 13 million kg were applied outside agriculture, for a total of 56.3 million kg (124 million pounds).

Global agricultural use of glyphosate mushroomed following adoption of GE-HT crops in 1996. The total volume applied by farmers rose 14.6-fold, from 51 million kg (113 million pounds) in 1995 to 747 million kg (1.65 billion pounds) in 2014 (Table 4). In this same time period, agricultural use of glyphosate in the U.S. rose 9.1-fold. Global non-agricultural uses have increased fivefold since the introduction of GE crops, from 16 million kg in 1995 to 79 million kg (35–175 million pounds; Table 4).

**Table 4:** Global agricultural and non-agricultural use of glyphosate: 1994 through 2014

	1994	1995	2000	2005	2010	2012	2014
Glyphosate use (1000 kg)	56,296	67,078	193,485	402,350	652,486	718,600	825,804
Agricultural	42,868	51,078	155,367	339,790	578,124	648,638	746,580
Non-agricultural	13,428	16,000	38,118	62,560	74,362	69,962	79,224
Glyphosate use (1000 lb)	124,112	147,882	426,561	887,030	1,438,485	1,584,242	1,820,585
Agricultural	94,508	112,608	342,525	749,108	1,274,546	1,430,002	1,645,927
Non-agricultural	29,604	35,274	84,036	137,922	163,940	154,240	174,658
Share agricultural (%)	76	76	80	84	89	90	90
Share non-agricultural (%)	24	24	20	16	11	10	10

Data in thousands of kilograms or pound of glyphosate active ingredient. See Additional file 1: Table S24 Table for details

Total worldwide glyphosate use (agricultural plus non-agricultural) rose more than 12-fold from about 67 million kg in 1995 to 826 million kg in 2014 (0.15–1.8 billion pounds; Table 4). Over the last decade, 6.1 billion kgs of glyphosate have been applied, 71.6 % of total use worldwide from 1974–2014 (Table 5).

**Table 5:** Share of total global glyphosate active ingredient use by decade

	Total use (million kg)	Increase from previous period	Share of total use 1974–2014 (%)
1974	3.2	NA	0.0
1975–1984	130.5	127	1.5
1985–1994	387.3	257	4.5
1995–2004	1909	1522	22.3
2005–2014	6133	4224	71.6
Total	8563		

Calculated from data in Additional file 1: Table S24

### Use on GE-HT crops

**Fig. 2:**
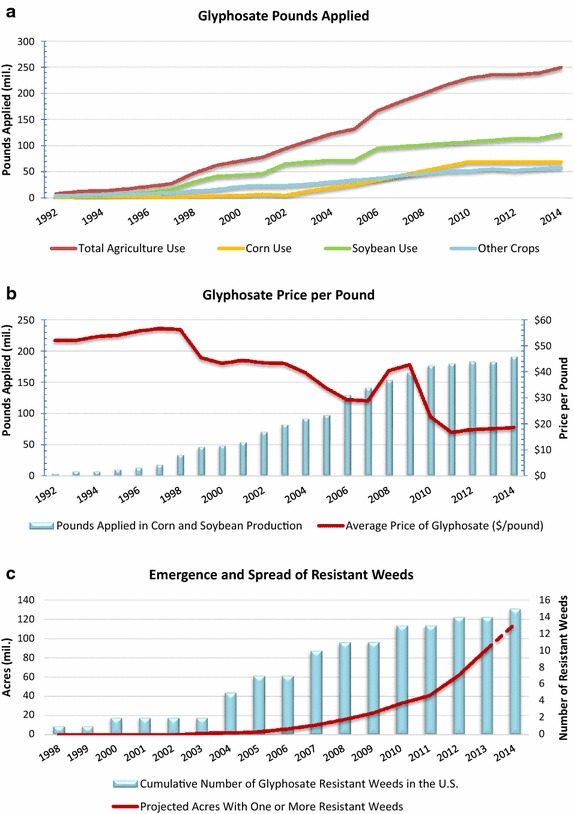
Use and impacts of glyphosate in corn and soybean production

For over a decade, the vast majority of hectares planted to maize, soybeans, canola, and cotton have been genetically engineered (GE) to be herbicide-tolerant (HT) (see Fig. 2a; [15–17]). In 2012, 265 million kgs of glyphosate were applied on GE-HT soybeans, or about 73 % of all glyphosate applied on GE-HT crops, and 41 % of total, global GBH use (Table 6). Between 2010 and 2012, glyphosate use rose moderately in GE-HT cotton production (10 %) and soybeans (19 %), but more sharply in GE-HT maize (47 %) and canola (36 %).

**Table 6:** Glyphosate use on herbicide-tolerant (HT) crops and all crops

	2010	2011	2012
Cotton	8.6	11.8	9.5
North America	5.64	6.99	6.32
Rest of world	3.00	4.8	3.1
Maize	47.7	65.6	70.2
North America	26.1	28.5	31.0
Rest of World	21.63	37.1	39.2
Soybeans	223.7	239.1	265.1
North America	41.9	42.0	43.6
Rest of world	181.7	197.1	221.5
Canola	13.7	16.5	18.6
North America	0.4	0.3	0.5
Rest of world	13.3	16.2	18.1
Global use on HT crops	293.7	333.0	363.4
Global use on All crops	578.1	616.8	648.6
Percent use on HT crops (%)	51	54	56

Data are millions of kilograms of glyphosate active ingredient

National Agriculture Statistic Service, International Service for the Acquisition of Agri-biotech Applications, and Meyer and Cederburg (2010). See Additional file 1: Table S23 for details

The percent of global agricultural glyphosate use accounted for by GE-HT crops rose from 51 % in 2010 to 56 % in 2012 (Table 6). This percentage cannot be calculated accurately for earlier years because comprehensive ISAAA time series data reporting on hectares planted to GE-HT crops began in 2010 [14].

## Discussion

### Volume applied in the U.S

The United States has the world’s most complete, publicly accessible data on glyphosate use. The combination of NASS, EPA, and USGS glyphosate use data provides a solid foundation to track trends in agricultural, non-agricultural, and total glyphosate use from commercial introduction through 2014. A report issued by the National Center for Food and Agricultural Policy [40] provides useful, detailed information on glyphosate use by state and crop for 1995, drawing on NASS, EPA, and information from land grant university weed management specialists.

Annual agricultural glyphosate use volumes in the nine EPA pesticide use reports issued between 1997 and 2007 exceed NASS annual totals for the same years by 20–70 %, largely because EPA had access to multiple data sources that made it possible to estimate the volume of glyphosate applied on all crops, as well as non-crop use patterns (e.g., pasture and range uses). NASS estimates, on the other hand, were limited in any given year to the crops surveyed in a particular year, and NASS never or rarely surveys pesticide use on crops grown on limited acreage. The differences are largest in the first two decades of glyphosate use (through 1995), and reflect the array of glyphosate uses not covered in NASS, crop-by-crop pesticide use surveys. But as total agricultural use rises sharply post-1996 in the wake of the introduction of GE-HT crops, glyphosate use on the major GE crops (maize, soybeans, cotton) is fully captured in NASS, EPA, and USGS data. Differences in agricultural use estimates between the datasets all but disappear by 2007 (NASS, 184.2 million pounds glyphosate use; EPA mid-range, 182.5; USGS, 183.2; [27], Additional file 1: Table S18).

### Factors driving use upward

Several factors have driven the increase in glyphosate use since commercial introduction in 1974. In terms of area treated, the dominant factor has been the commercialization of GE-HT crops. Not only has glyphosate been sprayed on more hectares planted to HT crops, it has also been applied more intensively—i.e., more applications per hectare in a given crop year, and higher one-time rates of application [13, 28].

In the U.S. soybean sector, the average number of glyphosate applications rose from 1.1 per crop year in 1996 to 1.52 in 2014, while the one-time rate of application rose from 0.7 kg/hectare (0.63 pound/acre) to 1.1 kg/hectare (0.98 pound/acre) in the same period ([27], Additional file 1: Table S2). Shifts in weed communities favoring species less susceptible to glyphosate, coupled with the emergence of first, less sensitive, and eventually glyphosate-resistant weeds drove the incremental rise in the intensity of glyphosate applications on GE-HT crops [13, 10]. Rising reliance on glyphosate by soybean producers in the U.S. is graphically portrayed in Fig. 1a, while Fig. 1b shows modest change during the GE era in soybean yield/acre or production per soybean seed planted. Steady increases in the number of applications of glyphosate, rate per crop year, and glyphosate’s share of overall soybean herbicide use are shown in Fig. 1c.

Other factors contributed to rising glyphosate use. These include steady expansion in the number of crops registered for use on glyphosate product labels, the adoption of no-tillage and conservation tillage systems, the declining price per pound of active ingredient (see Fig. 2b), new application method and timing options, and new agricultural use patterns (e.g., as a desiccant to accelerate the harvest of small grains, edible beans, and other crops).

The one-time average rate of glyphosate application on Kansas wheat has incrementally risen threefold, from 0.33 kg/hectare in 1993 to 0.95 kg/hectare in 2012 ([27], Additional file 1: Table S5). The trend toward no-till and conservation tillage systems has increased wheat farmer reliance on herbicides, including glyphosate. The average two applications in recent years on winter wheat could include a pre- or at-plant spray, an application during a summer fallow period, and/or a late-season application intended to speed up harvest operations (a so-called “harvest aid” or “green burndown” use) [41]. The average rate per crop year—the single most important indicator of the intensity of glyphosate use—rose even more dramatically, from 0.47 kg/hectare in 1993 to 2.08 kg/hectare in 2012 (4.4-fold).

Harvest-aid uses of glyphosate have become increasingly common since the mid-2000s in U.S. northern-tier states on wheat, barley, edible beans, and a few other crops, as well as in much of northern Europe [41–43]. Because such applications occur within days of harvest, they result in much higher residues in the harvested foodstuffs [42]. To cover such residues, Monsanto and other glyphosate registrants have requested, and generally been granted, substantial increases in glyphosate tolerance levels in several crops, as well as in the animal forages derived from such crops. Table 7 provides an overview of key crops on which regulatory authorities have granted large increases in glyphosate tolerances to accommodate GE-HT crop uses, as well as harvest aid, green burndown applications. Note the 2,000-fold increase in the glyphosate tolerance on dry alfalfa hay and silage from 1993 to 2014, an increase made necessary by the approval and planting of GE-HT alfalfa. In response to the large increase in expected residues from such uses, some European countries now prohibit harvest-aid applications on food crops (e.g., Germany, since May 2014).

**Table 7:** Changes in selected U.S. EPA glyphosate tolerance levels (ppm)

	1993	1999	2012	2015
Soybeans				
Grain	20	20	20	40
Hay	15	200	200	100
Forage	15	100	100	100
Maize				
Corn grain	0.1	0.1	5	5
Corn stover	NT	NT	6	100
Sweetcorn	0.2	0.2	3.5	3.5
Oats				
Grain	0.1	0.1	0.1	30
Wheat				
Grain	0.1	5	5	30
Straw	0.1	85	85	100
Edible beans	0.2	0.2	5	5
Alfalfa				
Dry hay	0.2	200	200	400
Silage	0.2	75	75	400

2012 and 2015 tolerances—40 CFR Part 180.364, “Glyphosate; tolerances for residues.” 1993 tolerances—”Glyphosate Reregistration Eligibility Document (RED),” (7508 W), Office of Pesticide Programs, U.S. EPA, September 1993. 1999 tolerances—EPA Tolerance Reassessment document for Reassessed Group 3 tolerances, August 4, 1999

### Global use of glyphosate

Farmers worldwide applied about 51.3 million kgs (113 million pounds) of glyphosate in 1995 ([27], Additional file 1: Table S23). To place this volume of global glyphosate use in perspective, in just one country (the U.S.) that year, farmers applied ~60 million kgs (132 million pounds) of two herbicides (atrazine and metolachlor) on mostly one crop (maize) ([27], Additional file 1: Table S19).

But the scope and intensity of glyphosate use worldwide rapidly changed as GE-HT crops gained market share. There were about 1.4 billion hectares of actively farmed, arable cropland worldwide in 2014 [44]. Across this landmass, there were an estimated 747 million kg of agricultural applications of glyphosate. Accordingly, if this volume of glyphosate had been applied evenly, about 0.53 kg of glyphosate could have been sprayed on every hectare of cropland on the planet (0.47 lbs/acre).

Glyphosate was, of course, not applied evenly on every hectare of cropland. The average rate of glyphosate applications per hectare per crop year during 2014 fell in the range of 1.5–2.0 kg/hectare [27]. At these rates of application, the total volume of glyphosate applied in 2014 was sufficient to treat between 22 and 30 % of globally cultivated cropland. No pesticide in history has been sprayed so widely.

Since losing global patent protection around 2000, dozens of companies began manufacturing technical glyphosate, and/or formulating glyphosate products. Some two-dozen Chinese firms now supply 40 % of the glyphosate used worldwide, and export most of their annual production [45].

The loss of patent protection and increased generic manufacturing of glyphosate has placed downward pressure on prices since 2000 [30, 45, 46]. The major manufacturer, Monsanto, has typically not competed directly or solely on price, and instead has been successful in holding or expanding market share by bundling purchase of higher-price, Monsanto brand, Roundup herbicides with the purchase of Monsanto herbicide-tolerant seeds [45–47]. Especially in the U.S., this bundling strategy has been augmented by various volume incentives and discounts, special financing, rebates for purchase of other herbicides working through a mode of action other than glyphosate’s (to delay the spread of resistant weeds), and other non-price benefits tailored to appeal to large volume customers [46–48].

The diversity of global uses in agriculture and other sectors has grown over the past 40 years [9], making it more difficult to compile accurate global data across all glyphosate uses, especially by sector and specific use. As a result, global glyphosate use projections can only be based on industry-wide glyphosate production figures, as done from 1997–2014 in Table 4 and Additional file 1: Table S24 [27].

### Impact of GE-HT technology

The development and marketing of GE, Roundup Ready crops fundamentally changed how crop farmers could apply glyphosate. Before RR technology, farmers could spray glyphosate prior to crop emergence, for early-season weed control, or after harvest to clean up late-season weeds. But with RR crops, glyphosate could also be sprayed 1–3 times or more after the crop had emerged, leaving the crop unharmed but controlling all actively growing weeds. This historically significant technological advance set the stage for unprecedented and rapid growth in the area planted to RR crops and sprayed with glyphosate (from usually less than 10 % of cotton, maize, and soybean acres pre-1996, to 90 % or more today) [47, 49, 50].

The interplay of various factors leading to increased glyphosate use is apparent in Fig. 2a, which shows the trend in overall glyphosate use on the key GE-HT crops in the U.S., the correlation between reductions in average price per pound and use (Fig. 2b), and rising use and the emergence of resistant weeds (Fig. 2c).

Use of glyphosate on some GE-HT crops may have declined, or may soon begin declining in some regions because (a) adoption of GE-HT soybeans, cotton, and canola has peaked in most of the countries that have embraced GE technology [9], and (b) farmer willingness to pay for repeat applications of glyphosate, or further increase application rates, typically declines as glyphosate-resistant weeds become well established, as they have in much of the U.S. [13] and in Brazil and Argentina [10]. On the other hand, GE-HT crops may move into some regions not previously planting them (e.g., China), and reductions in the price of generic glyphosate herbicides could lead to more intensive use in some countries.

In the countries that have planted the largest shares of GE-HT crops (the U.S., Argentina, and Brazil), glyphosate use rates per hectare per crop year have risen sharply since around 2000 [20, Additional file 1: Tables S2, S3, S22]. Worldwide on GE soybean and cotton, average total herbicide use per crop year per hectare has approximately doubled from 1996 to 2014, with the increase in glyphosate volumes applied per hectare accounting for nearly all of the per hectare increase. Maize herbicide use per hectare has risen modestly, if at all, in large part because adoption of GE-HT maize hybrids allowed farmers to reduce reliance on a half-dozen other widely used maize herbicides applied at relatively high rates (e.g., ~1 kg/hectare per crop year) [11].

Because GE-HT soybeans account for two-thirds of the total hectares planted to GE-HT crops worldwide, the doubling of average herbicide use per hectare of HT soybeans drives the sizable increase in overall herbicide on all GE crop hectares. There is, as well, a clear connection throughout South America in the adoption of GE-HT technology and no-tillage systems [17, 38]. No-till farming in South America lowers machinery and labor costs, and reduces soil erosion, but at the expense of heightened reliance on herbicides for weed control, and other pesticides to control insects and fungal pathogens.

Despite gaps in publicly accessible data, the dramatically upward trajectories in glyphosate use in the U.S. and globally are unmistakable. In the pre-GE era (1974–1995) in the U.S., non-agricultural glyphosate uses accounted for ~34 to 42 % of total use. The share of total glyphosate use accounted for by the agricultural sector shifted markedly upward post-1996, starting at 66 % in 1996 and reaching 81 % 5 years later (2001) and 92 % by 2014 ([27], Additional file 1: Table S18).

The total volume of use and the split between agricultural and non-agricultural uses in the pre-GE era period are subject to greater uncertainty than in the 1996–2014 period. However, pre-1995 glyphosate use is minor compared to the post-GE period, when both data quantity and quality improved, especially covering applications in the U.S. and on global GE-HT hectares planted.

**Fig. 3:**
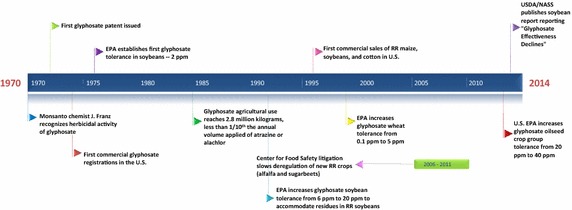
Milestones in the history of glyphosate discovery, commercialization, and regulation

**Fig. 4:**
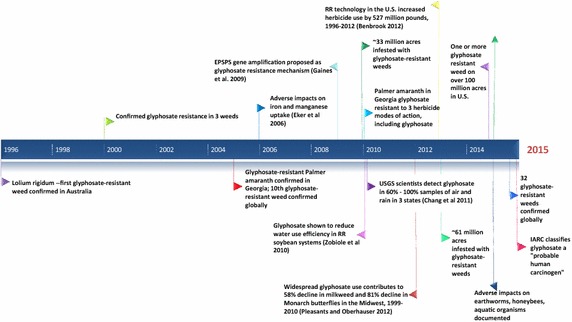
Milestones in glyphosate use and impacts

Figure 3 arrays milestones in the history of glyphosate discovery, commercialization, and regulation, while Fig. 4 displays key events in the history of glyphosate use and impacts.

### Rising use triggers new concerns

Driven by the growing diversity of uses and dramatic increases in volumes applied, levels of glyphosate and its primary metabolite aminomethylphosphonic acid (AMPA) have been detected in the air [51], soil [52], and water [49, 53]. With few exceptions though, contemporary levels of glyphosate in the air, water, and food result in typical human exposure estimates that remain well below the “levels of concern” or “Acceptable Daily Intakes” established by regulatory bodies around the world.

Still, a growing body of literature points to possible, adverse environmental, ecological, and human health consequences following exposure to glyphosate and/or AMPA, both alone [54] and in combination with ingestion of GE proteins (e.g., EPSPS, *Bt* endotoxins) [55]. Environmental studies encompass possible glyphosate impacts on soil microbial communities and earthworms [56–58], monarch butterflies [59], crustaceans [60], and honeybees [61].

Studies assessing possible risks to vertebrates and humans include evidence of rising residue levels in soybeans [62, 63], cancer risk [64], and risk of a variety of other potential adverse impacts on development, the liver or kidney, or metabolic processes [54, 55, 65–80].

### Relative toxicity and impacts

For years, glyphosate has been regarded as among the least chronically toxic herbicides for mammals, and indeed only three EPA-registered synthetic pesticides in current agricultural use have a higher chronic Reference Dose (the imidazolinone herbicides imazamox, imazethapyr, and imazapyr).

For human exposures, the U.S. EPA has set glyphosate’s daily chronic Reference Dose (cRfD) at 1.75 milligrams per kilogram of bodyweight (mg/kg bodyweight/day). The EU-set cRfD for glyphosate was recently raised from 0.3 to 0.5 mg/kg/day, 3.5-fold lower than EPA’s. A team of scientists has compiled evidence supporting the need for a fivefold reduction in the EU cRfD to 0.1 mg/kg/day [81], a level 17-times lower than EPA’s.

Glyphosate is a moderate dose herbicide with relatively low acute and chronic mammalian toxicity, to the extent mammalian risk is accurately reflected in required EPA toxicology studies. After an exhaustive review, however, glyphosate was classified in 2015 as a “probable human carcinogen” by the International Agency for Research on Cancer [64], based on increased prevalence of rare liver and kidney tumors in chronic animal feeding studies, epidemiological studies reporting positive associations with non-Hodgkin lymphoma, and strong mechanistic evidence of genotoxicity and ability to trigger oxidative stress [64].

The body of toxicological studies supporting glyphosate’s current EPA and EU cRfD, and hence all contemporary uses of this herbicide, dates back to the early 1970s through mid-1980s [82]. Recent studies suggest that glyphosate in its pure form, and some formulated glyphosate end-use products, may be triggering epigenetic changes through endocrine-mediated mechanisms [54, 73, 75, 76, 79, 81, 83].

Evidence from multiple studies suggests that the kidney, and secondarily the liver, is at risk of glyphosate-triggered, or glyphosate-enhanced chronic degeneration [55, 71, 72, 84, 85]. Industry metabolism studies in farm animals, rats and mice, and rabbits were conducted in the 1970s and 1980s, and show that in animal feeding studies, glyphosate levels in the kidney usually exceed those in the liver by three- to tenfold, and those in the liver exceed levels in other tissues by a wide margin [86].

The apparent tendency of glyphosate to concentrate in the kidneys, coupled with glyphosate’s action as a chelating agent, has led some scientists to hypothesize that glyphosate can bind to metals in hard drinking water, creating metallic-glyphosate complexes that may not pass normally through kidneys [71, 72]. For this, or other as yet unrecognized reasons, the risk of chronic kidney disease may be heightened in human and animal populations with heavy glyphosate exposure.

The IARC classification and emerging evidence relative to kidney damage and endocrine effects heightens the need for, and will complicate ongoing and future glyphosate worker and dietary-risk assessments. Annual residue tests are carried out by the U.K. Food Standards Agency (FSA). Residues of glyphosate were found in 10–30 % of grain-based samples from 2007–2013, at generally rising levels [87]. Glyphosate and AMPA residues are present at relatively high, and rising levels (over 1 ppm) in a high percentage of the soybeans grown in the U.S., Canada, Brazil, Argentina, Paraguay, countries which account for 86.6 % of the 11.6 billion bushels of soybeans produced globally in 2014, and nearly all global trade in soybeans and soybean-based animal feeds [34, 62].

## Conclusions

A high level of confidence can be placed in the trends in glyphosate use in the U.S. because of consistency across three independently compiled datasets (USDA-NASS, EPA, and USGS).

A published paper by a pesticide industry consultant provides solid data on global glyphosate use in 1994–1997, both in the agricultural and non-agricultural sectors [29]. Lack of publicly accessible data on global glyphosate use since the mid-1990s increases the uncertainty in the global estimates reported herein. However, since the majority of the increase in global glyphosate use since the late-1990s was driven by the adoption of GE-HT crops, accessible data from ISAAA and the literature on GE-HT crops provide a solid basis to project total glyphosate use on GE-HT crops over the last ~15 years.

By any measure, glyphosate-tolerant crop technology has been an enormous commercial success, and at least initially, simplified weed management in maize, soybean, and cotton crops both in the U.S. and worldwide [2, 9, 88]. For a few years post-1996, one, or at most two applications of glyphosate proved highly effective and economical on nearly all cropland planted to GE-HT seeds. As a result, the land area treated with glyphosate rose rapidly. Over time this triggered the emergence of weed phenotypes less sensitive or resistant to glyphosate. In response, farmers increased both the rate of glyphosate application as well as the number of applications [5, 6, 9, 88, 13]. Many farmers also integrated additional herbicides into spray programs [5–7, 89]. As a direct result, average herbicide use per hectare on land planted to GE-HT varieties has, on average, escalated steadily since the mid-1990s [8, 11, 88, 13].

The upward trend in glyphosate use has, and will likely continue to contribute to incremental increases in environmental loadings and human exposures to glyphosate, its major metabolite aminomethylphosphonic acid (AMPA), and various surfactants and adjuvants used in formulating end-use glyphosate-based herbicides.

Given that glyphosate is moderately persistent and mobile, levels in surface and groundwater will likely rise in step with use, and this will increase the diversity of potential routes of animal and human exposure.

Human exposures from around the home and urban uses of glyphosate also warrant closer attention. Most end-use, glyphosate products sold for home and urban use in developed countries contain relatively low concentrations of glyphosate, so the risk of experiencing an acutely toxic exposure is minimal. But in developing countries, risks stemming from applications of more concentrated glyphosate products and/or applications of “home-mixed” products should not be ignored.

The frequency and levels of glyphosate and residues in a variety of foods are increasing, and more refined dietary-risk assessments should be carried out. Reasonably accurate estimates of glyphosate residues and dietary exposures in areas lacking residue data can be made drawing on insights gained from risk assessments conducted in areas with accurate glyphosate use and residue data.

## Additional file

10.1186/s12302-016-0070-0 List of supplemental tables.

## Abbreviations

AMPA: aminomethylphosphonic acid
Bt: Bacillus thuringiensis
EPA: Environmental Protection Agency
GBH: glyphosate-based herbicide
GE: genetically engineered
GE-HT: genetically engineered herbicide-tolerant [crop]
GMO: genetically engineered organism
HT: herbicide tolerant
Kg: kilogram
NASS: National Agricultural Statistics Service (a USDA agency)
RR: Roundup Ready
USDA: United States Department of Agriculture
USGS: United States Geological Service
